# Yield Sensitivity of Mungbean (*Vigna radiata* L.) Genotypes to Different Agrivoltaic Environments in Tropical Nigeria

**DOI:** 10.3390/plants14091326

**Published:** 2025-04-28

**Authors:** Uchenna Noble Ukwu, Onno Muller, Matthias Meier-Gruell, Michael Ifeanyi Uguru

**Affiliations:** 1 Department of Crop Science, University of Nigeria Nsukka, Nsukka 410001, Enugu State, Nigeria; michael.uguru@unn.edu.ng; 2 Institute of Bio-and Geosciences (IBG-2), Plant Sciences, Forschungszentrum Julich GmbH, 52425 Julich, Germany; ma.meier@fz-juelich.de

**Keywords:** Agri-photovoltaics, Finlay and Wilkinson analysis, genotype × environment interaction, AMMI, PV orientation, stability

## Abstract

Genotype by environment (G × E) interaction is a magnitude change in the performance of a genotype when grown in contrasting environments. The sensitivity of a genotype to different environmental conditions is an important determinant of its suitability for cultivation in a specific environment or across multiple environments. In many nations of the world, where the drive to achieve a net-zero CO_2_ emission by 2030 has spurred significant investments in clean energy sources such as photovoltaics with a resultant conversion of some agricultural lands to photovoltaic facilities, there is a need to find the right balance between addressing the food and energy crises. Agri-photovoltaics (APV) offer a sustainable solution by allowing crops to grow underneath photovoltaic panels. However, selection efficiency and repeatability of APV experimental results could be marred by the presence of G × E interaction. The study objective was to identify mungbean genotype(s) with a high yield potential and broad adaptability across APV environments. Five mungbean (*Vigna radiata* L.) genotypes, Tvr18, Tvr28, Tvr65, Tvr79, and Tvr 83, were assessed under six contrasting APV environments, EPV-R, EPV-D, NPV-R, NPV-D, WPV-R, and WPV-D, at the Agri-PV Food and Energy Training Center, University of Nigeria, Nsukka. The experiment was a split-plot design, with the environment as the whole-plot factor while genotype was the sub-plot factor with five replications. The additive main effects and multiplicative interaction (AMMI) and the Finlay and Wilkinson joint regression analysis confirmed significant genotype, environment, and G × E interaction effects for mungbean seed yield. Two genotypes, Tvr28 and Tvr83 expressed broad adaptability to the APV environments with higher yields (2.60 and 2.50 t ha^−1^), ranking first and second, respectively. In contrast, the Tvr79 genotype displayed the highest sensitivity (2.95) to environmental variation and was unstable across the environments with higher IPCA1 and ASV scores of −1.17 and 1.39, respectively. The EPV-R recorded the highest yield (2.61) with low interaction effect (0.38), whereas the WPV-D environment had the least yield (1.71) and was the most unstable (−0.48). Conclusively, the Tvr28 and Tvr83 genotypes and the EPV-R environment were the ideal genotypes and environment, respectively, and are therefore recommended for use in APV facilities.

## 1. Introduction

Genotype by environment (G × E) interaction is a magnitude change in the performance of a genotype when grown in contrasting environments [1]. It could sometimes result in a change in ranking order of a genotype when tested in multiple environments [2]. The sensitivity of a genotype to different environmental conditions is an important determinant of its suitability for cultivation in a specific environment or across multiple environments [3]. In many nations of the world, where the drive to achieve a net-zero CO_2_ emission by 2030 has spurred significant investments in clean energy sources such as photovoltaics, with a resultant conversion of some agricultural lands to photovoltaic facilities [4,5,6,7], there is a need to find the right balance between addressing food and energy crises.

Agri-photovoltaics (APV) offer a sustainable solution by allowing crops to grow underneath photovoltaic panels. This approach help to balance future food and energy demands [8], optimizes land use [9], reduces excess solar radiation [10,11,12], and improves photochemical efficiency [8,13] of a crop. Additionally, APV-generated electricity can power farm operations, while the shading effect can reduce water loss during dry periods, leading to cost savings. Yet, selection efficiency and repeatability of experimental results underneath APV environments could be affected by large G × E interaction effect [2].

Mungbean seeds are very rich in major food nutrients, containing about 30% of crude protein, 45% of carbohydrate, 6.5% of fiber, and some essential amino acids [14]. The crop has therapeutic and curative properties in varied health related diseases due to high levels of phytochemicals like vitexin, isovitexin, total phenols, and flavonoids contained in the leaves and seeds [15]. Considerable variability exists among mungbean germplasm, especially in morphological traits like plant height, pod length, number of pods per plant, and seed yield [12]. Mungbean (*Vigna radiata* L.) is rapidly gaining interest as a crop in major tropical and subtropical regions of the world due to its high nutritional content. It is a staple food and an income source for many households in South and Southeast Asia. In the last ten years, several studies have been conducted on mungbean in several parts of Africa [16], further emphasizing its growing importance. While environment play a crucial role in a crop’s physiological processes such as photochemical energy conversion, respiration, and nutrient absorption, G × E is a key factor influencing phenotypic expression in multi-environment trials [3]. The phenotype of a crop is a composite of the genotype and environment main effects, and the G × E interaction effect [17,18]. G × E is an integral component in breeding a crop for wide environment adaptability [19], because a large G × E interaction term could mask the gains in a selection program and reduce the likelihood of identifying superior genotypes [20]. The stability of genotypes across multiple environments has constituted a serious challenge to plant breeders for several years because it reduces the selection efficiency and the repeatability of experimental results in different environments [2]. Therefore, from a breeding perspective, it is desirable for a genotype to demonstrate high stability in different environments in order to maintain its desirable traits and productivity. Such stability conserves resources and enhances the predictability of crop performance. An ideal genotype should demonstrate both stability and broad adaptability across different conditions. Plant breeders analyze G × E interaction effect in order to determine the proportion of variation that can be predicted versus what remains unpredictable. Three commonly used statistical models for G × E analysis are the additive main effects and multiplicative interaction (AMMI) model, the genotype main effects and G × E interaction (GGE) model, and the Finlay and Wilkinson joint regression approach [2,21,22,23,24]. The objective of this study was to identify genotype(s) with high yield potential and stability in APV environments across seasons. This study is the second report of genotype by environment interaction and yield stability analysis within an APV facility globally.

## 2. Results and Discussion

### 2.1. AMMI Analysis

The result of the AMMI ANOVA showed a highly significant effect (*p* < 0.001) of genotype, environment, G × E interaction, and the interaction principal component axis (IPCA 1 and IPCA 2) (Table 1). The analysis indicated that the treatments explained about 83% of the total sum of squares variation in contrast to error which explained about 15%. Out of the 83% of the treatment effects, genotype and G × E interaction exerted greater effects on mungbean seed yield with a combined effect of over 83% (45.5 and 37.9, respectively) than environment (17%). Overall, the genotype accounted for the largest percentage of the treatment effect (45%), an indication that at least one of the five mungbean genotypes was clearly distinct from the rest. The G × E interaction component had the next highest treatment effect on seed yield (38%), suggesting that at least one genotype responded differently across the various environments. The environment exerted the least effect on mungbean seed yield (17%), suggesting that at least two environments were similar. The AMMI ANOVA further partitioned the significant G × E interaction variance into IPCA1 and IPCA2, which explained 52% and 44% of the interaction variance, respectively, with the residual making up the remaining 4% (Table 1). This report is coherent with findings by Mattos et al. [17], Regis et al. [18], and Tena et al. [24], who reported that the greater percentage of the G × E interaction effect was accounted for by IPCA1 and IPCA2.

The yield scores for genotype, environment, G × E interaction, IPCA1, and IPCA2 are shown in Table 2. In addition, the ASV scores of the five mungbean genotypes underneath six APV environments were also shown. The yield scores of genotype and environment are contained in rows and columns, respectively. The genotype Tvr18 recorded the highest yield score (2.62), followed by Tvr28 (2.60), while Tvr65 was the poorest (1.27). Three genotypes, Tvr18, Tvr28, and Tvr83, recorded above average seed yield (>2.23 t ha^−1^), while Tvr65 and Tvr79 recorded below average yields (<2.23 t ha^−1^) and were poor. The EPV environments ranked first (2.65) and third (2.25) in the rainy and dry seasons, respectively. The NPV environments ranked second (2.50) and fifth (2.02) in rainy and dry seasons, respectively, while the WPV environments ranked fourth (2.23) and sixth (1.71) and were the poorest. The superior seed yield recorded underneath the EPV environments in contrast to the NPV and WPV environments could be explained by the improved environmental conditions provided by the EPV environment. In particular, the reduced intercepted PAR and temperature, and the improved air humidity, encouraged the efficient utilization of water and assimilates for photochemical energy production rather than for non-photochemical heat dissipating reactions like excessive evapotranspiration and accumulation of reactive oxygen species that could potentially damage photosynthetic pigments like the D1 protein under excess sunlight. The shade provided by the EPV environment enabled the crops to maintain a more stable and optimal environment for plant growth. Consequently, enhanced physiological processes led to a resultant higher yield underneath the West–East-oriented APV environment across the two seasons. This report is in alignment with Ukwu et al. [12], who reported enhanced growth and photosynthesis performance of mungbean crops underneath the EPV environment compared to the WPV.

The IPCA1 represents the most important genotype by environment interaction effects and explains the largest proportion of the G × E interaction while IPCA2 explains the second largest G × E interaction term. High IPCA scores indicate strong interactions while low or near-zero IPCA scores show weak interactions. The IPCA scores of the genotypes revealed that Tvr79 recorded the highest interaction score in IPCA1 (−1.17), while Tvr18 had the highest score (1.04) in IPCA2. The other genotypes Tvr28, Tvr65, and Tvr83 recorded IPCA scores less than 0.60 in both IPCA1 and IPCA2 and were less responsive to the different environments. Interestingly also, the ASV scores followed a similar trend with Tvr65, Tvr83, and Tvr28 recording 0.53, 0.61, and 0.69, respectively. ASV is a very important index used by plant breeders to select genotypes that are stable across multiple environments. A stable genotype has a higher predictability index than an unstable genotype. ASV scores close to zero infer high stability.

Consequently, Tvr28 (0.61) and Tvr83 (0.69), which recorded low and near-zero ASV scores in addition to scoring above-average seed yield, were the most stable genotypes, whereas Tvr18 was unstable even though it recorded the highest yield. ASV is widely recognized as an effective method for assessing genotype stability and has been widely used alongside other stability metrics like Eberhart and Russel, Finlay and Wilkinson, Shukla, and Wrick [24]. A stable genotype is a genotype that records above-average yield and is less interactive when grown in multiple environments. The significant G × E interaction for mungbean seed yield indicates that both genotype and environment influenced yield variation, with neither factor alone explaining it. G × E interaction affects the outcome of any selection process and is an important component of any plant breeding program [25]. The variable responses of genotypes across the APV environments highlight the importance of selecting stable and well-adapted genotypes for APV systems. Although a low G × E interaction effect is desired by a plant breeder for stability across multiple environments, a high G × E can be advantageous when breeding for a specific environment [26]. It is common knowledge that plant breeding thrives on the existence of genetic variability. As such, obtaining a correct estimate of genotypic variance is only possible if such estimates are unmasked by variation arising from G × E interaction variance [2] because the use of inaccurate genotypic estimates could result in a total waste of time and effort.

**Figure 1:**
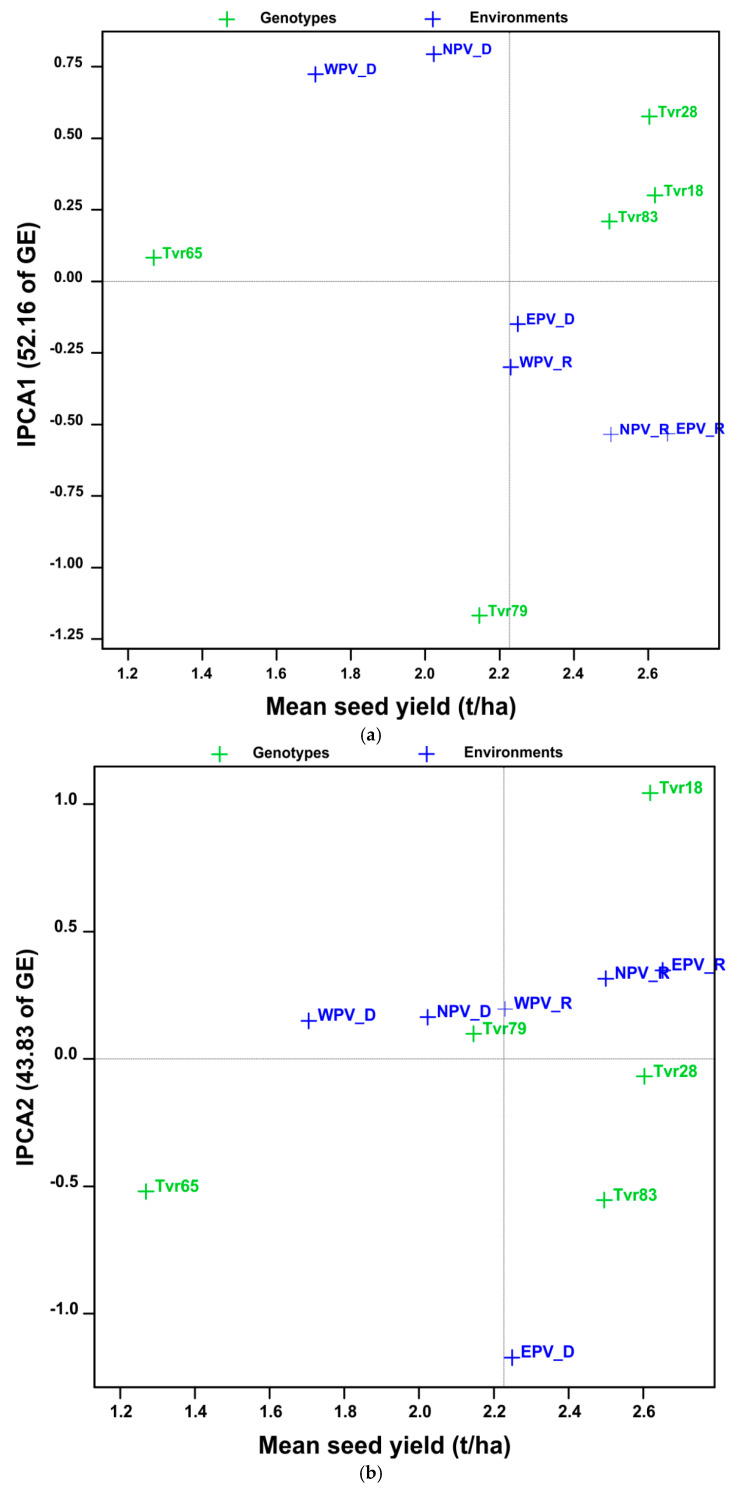
(**a**) Biplot of seed yield vs. IPCA1 for five mungbean genotypes in six APV environments. (**b**) Biplot of seed yield vs. IPCA2 for five mungbean genotypes in six APV environments.

The AMMI biplot in Figure 1a is a diagrammatic illustration of the genotype and environment scores on the x-axis and the IPCA1 scores on the y-axis. Treatments of a factor that are positioned close to a vertical line exhibit similar main effects, while those near a horizontal line demonstrate comparable interaction patterns [27,28]. Accordingly, the EPV-D and WPV-R environments were located close to the vertical line and were comparable in yield. Similarly, Tvr28 and Tvr18, which were aligned vertically, also had comparable yield. The NPV-R and EPV-R environments which recorded higher yield scores also showed weak interaction patterns in IPCA1 (Figure 1a). The vertical line separated the treatments into two halves, below average performers (left) and above average performers (right). The biplot diagram further reiterates the superior yield quality of Tvr18 and Tvr28 as variables located the farthest right on the x-axis are the best performers. Hence, Tvr65 and Tvr79 genotypes and WPV-D and NPV-D environments were poor performers. Research has shown that large IPCA1 scores, whether positive or negative, indicate strong interactions, whereas scores near zero signify weak interactions. In general, IPCA1 explained 52.2% of the G × E interaction term, whereas IPCA2 explained 43.8%. Except for EPV-D, which was highly unstable with a large interaction score in IPCA2, the other five environments had small interaction patterns aligning along the horizontal axis (Figure 1b). However, only two environments, EPV-R and NPV-R were desirable having scored above average seed yield. Likewise, Tvr28 was the most stable genotype in IPCA2. Overall, there was inconsistency in the performance of genotypes across the mega-environments. Environment IPCA1 and IPCA2 scores had both negative and positive values (Table 2) implying that there was a difference in ranking orders among genotypic yield performances across environments.

### 2.2. Mean Performance and Stability of Mungbean Genotypes and APV Environments by GGE Biplot

**Figure 2:**
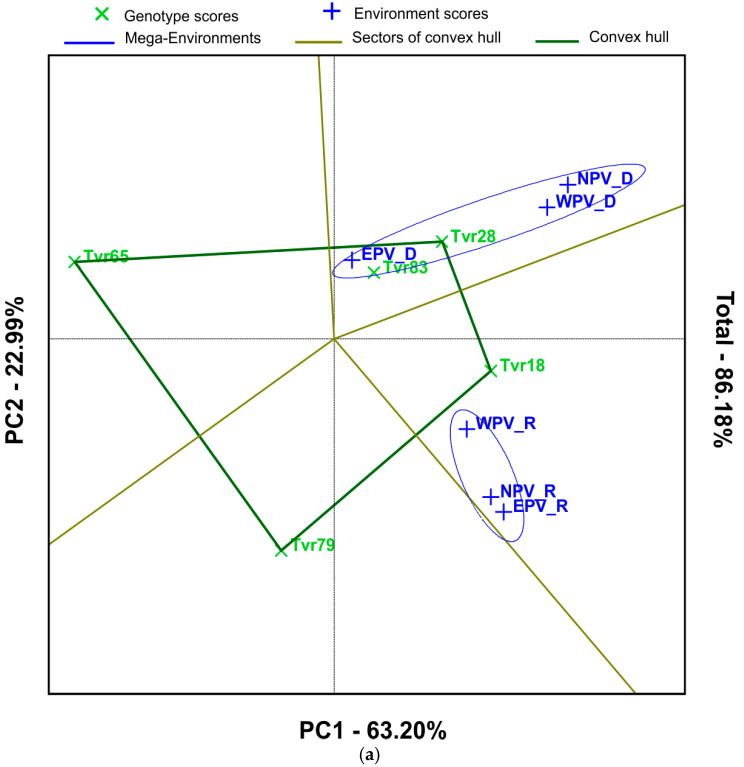

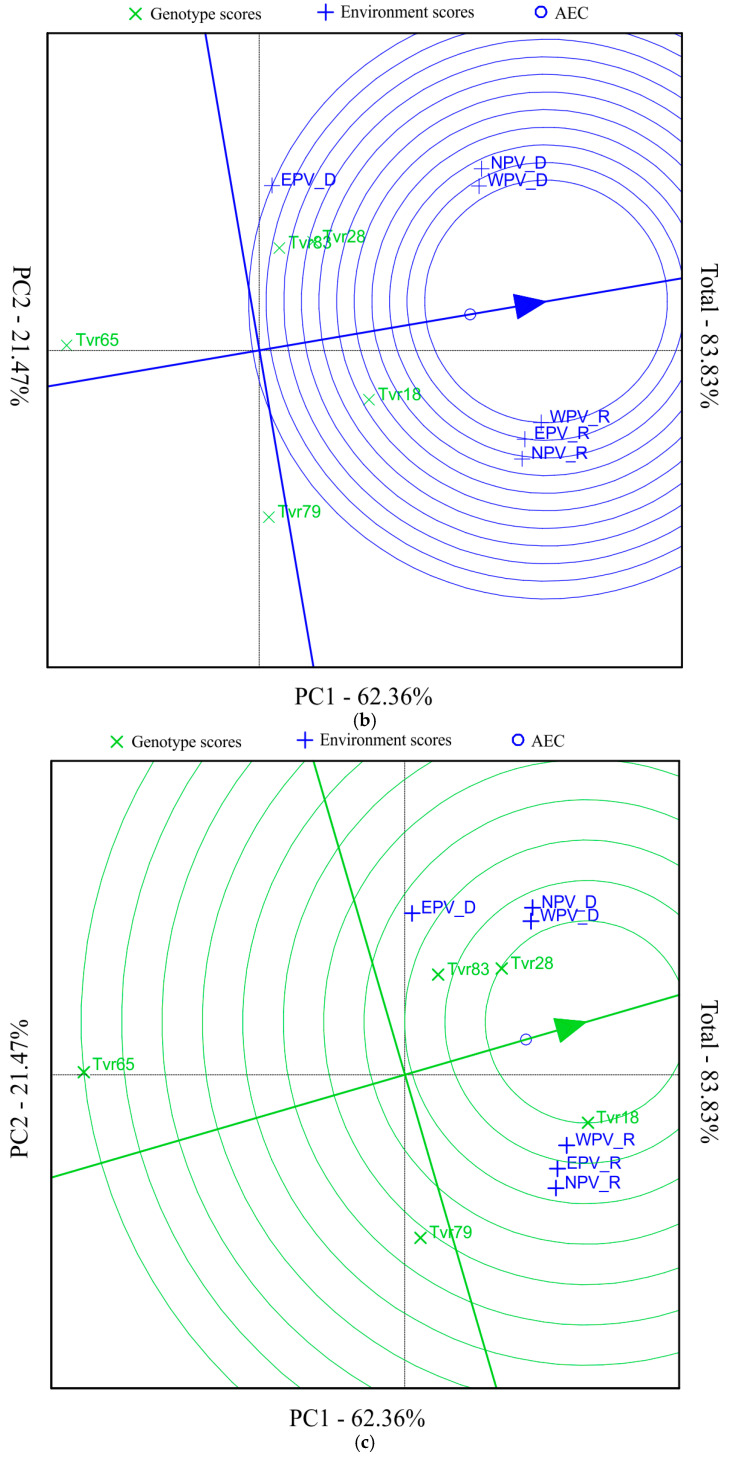
(**a**) GGE biplot analysis of seed yield showing the “which-won-where” view of five mungbean genotypes in six APV environments. (**b**) Comparison of environments by the GGE-biplot based on environment-focused scaling for identification of the ideal environment. (**c**) Comparison GGE biplot discriminating the genotypes with the above average yield from the genotypes with below average yield across the APV environments.

The “which-won-where” perspective, which offers a concise summary of the G × E interaction pattern of a multi-environment yield trial data set, is shown in Figure 2a. The vertex genotypes (Tvr18, Tvr28, Tvr65, and Tvr83) were connected together to form a polygon. The vector length and direction indicate the degree of the genotypes’ response to the APV environments. A series of lines that intersected the polygon’s sides and were drawn from the biplot origin separated the biplot into four sectors. Additionally, two mega-environments were created from the biplot. Mega-environment 1 comprised all the environments in the dry season (EPV-D, WPV-D, and NPV-D), whereas mega-environment 2 consisted of all environments in the rainy season (EPV-R, WPV-R, and NPV-R). In a mega-environment, the genotypes on the vertices of the polygon are the best-performing genotypes in those environments for the trait under investigation [29], while genotypes that are farthest from the origin are unstable. Consequently, Tvr28 and Tvr83 genotypes were the best in mega-environment 1, while Tvr18 and Tvr79 were the best in mega-environment 2. Tvr65 located at the left vertex of the polygon had very poor performance in all the environments.

The GGE biplot explained 86.02% of the variation. Based on the GGE biplot analysis, Tvr83 and Tvr28 were the most stable across the APV environments having recorded the shortest distance from the origin. Additionally, the GGE biplot also revealed that mega-environment 2 (rainy season) favored higher seed yield than mega-environment 1 (dry season). This finding was corroborated by Regis et al. [18] and Tena et al. [24].

The AEC method was also used to assess the yield stability of genotypes (Figure 2b). A blue line connecting the ideal environment to the biplot origin highlighted the genotypes with the highest main effects. The greater the distance between a genotype and the AEC coordinate, the greater the G × E interaction, and the lower the genotype stability. The GGE comparison biplot (Figure 2b,c) was used to screen the ideal environment (Figure 2b) and the ideal genotype (Figure 2c). The blue and green arrows point to the concentric circles with the ideal environment(s) and genotype(s), respectively. An ideal environment is an environment that allows the full expression of genotypes by providing optimal conditions for growth, development, and productivity. An ideal genotype on the other hand is one with the highest mean performance and is stable [29]. It should have a high mean yield across most environments. With regard to concentric circles, variables occupying the last concentric circle for any case are the ideal treatment. Therefore, the WPV-R environment occupying the last concentric circle in Figure 2c was identified as the ideal environment, whereas EPV-D positioned at the first concentric was the most discriminative or unstable environment. Likewise, the genotypes Tvr28 and Tvr18 were identified as the ideal genotypes (Figure 2c), whereas Tvr65 was the poorest. The AEC coordinates also partitioned the genotypes with the greatest main effects (Tvr18, Tvr28, and Tvr83) from the genotypes with below-average effects (Tvr65 and Tvr79).

**Figure 3:**
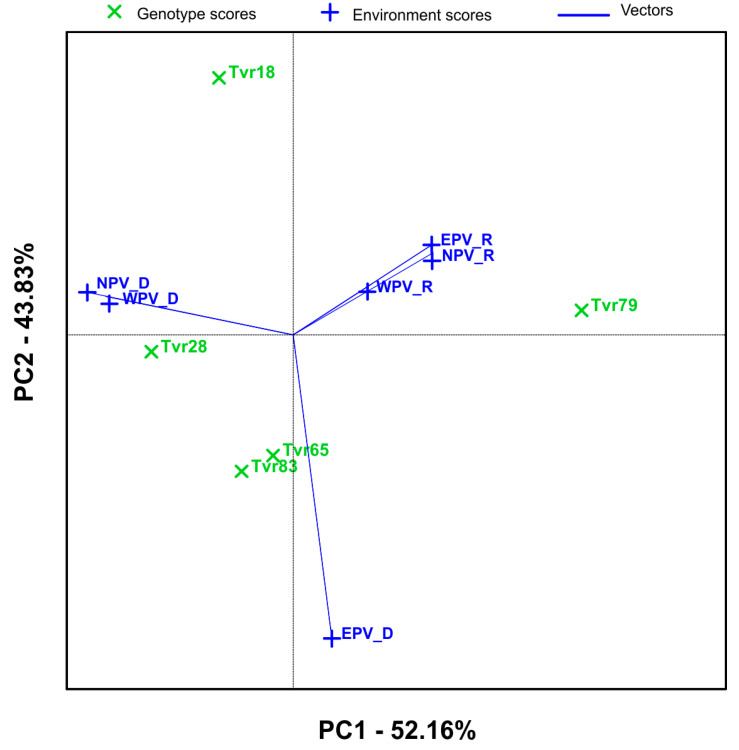
AMMI biplot showing correlations among environments based on symmetric scaling for environments.

The correlation among the environments is shown in the vector view of the AMMI biplot (Figure 3). The angle between the environment vectors defines the type of relationship between any two environments. An acute angle (<90°) indicates a positive correlation; an obtuse angle (>90 and <180) indicates a negative correlation while a right angle indicates no correlation. Hence, Figure 3 showed that EPV-R, NPV-R, and WPV-R were positively correlated. NPV-D and WPV-D were also positively correlated, whereas EPV-D was negatively correlated with the other five environments (Figure 3). In general, the rainy season environments were negatively correlated with the dry season environments implying high environmental sensitivity of the mungbean genotypes in terms of seed yield. Hence, a genotype that excelled in one of the APV environments in the rainy season may not perform well in the same environment in the dry season. Although most researchers have reported positive correlations between test environments, negative correlations have also been reported by Mattos et al. [17] and Tena et al. [24].

The differential responses of the mungbean genotypes to the contrasting APV environment are a consequence of the differences in their genetic constitution and preference for shade or reduced light conditions which had previously been reported by Ukwu et al. [12,30,31,32]. The variation due to environment could be implicated in the effect of the PV modules in moderating the microclimate of the APV environments. Microclimate indices of a plant such as PAR, temperature, and relative humidity exert considerable influence on crop growth and development. Crops perform optimally under specific climatic conditions, and any sharp deviation from the optimal could greatly affect crop productivity. PAR and temperature were decreased under WPV and EPV environments compared to the control (no PV shading) environment for most part of the study duration both in the rainy and dry seasons due to the effect of the PV modules in shading off between 5 and 250% of incident radiation, consequently exerting a cooling effect with reduced evapotranspiration. The variation between the performance of the APV environments, WPV and EPV, could have arisen from the direction of the PV panels. Although the same PV panels were used, the PV direction was different, which emphasizes the significance of panel orientation when installing an APV facility. This study recorded higher PAR and temperature values with lower relative humidity under the EPV compared to the WPV in the dry season which could be attributed to the rising of the sun from the East and setting at the West, implying that the EPV had more sun hours compared to the WPV. In the rainy season however, there was no clear trend as PAR and temperature were higher in the EPV environment at 5, 7, and 8 WAP and lower than the WPV environment at 4 and 6 WAP. In general, the APV environments in the rainy season provided better environmental conditions for the mungbean plants which enhanced higher average yield (2.46 t ha^−1^) compared to the dry season (1.99 t ha^−1^).

Additionally, the Finlay and Wilkinson joint regression analysis of variance (Table 3) further confirmed that the genotype had the greatest effect on mungbean yield, accounting for about 37% of the total variation. The environmental effect was also significant (*p* < 0.0001) and accounted for about 14% of the total variation. The significant sensitivity index of the Finlay and Wilkinson ANOVA table suggests that at least one of the genotypes responded differently in at least one of the APV environments (Table 3) due to a large G × E interaction. This report is coherent with the report of Banik et al. [33] on maize.

**Figure 4:**
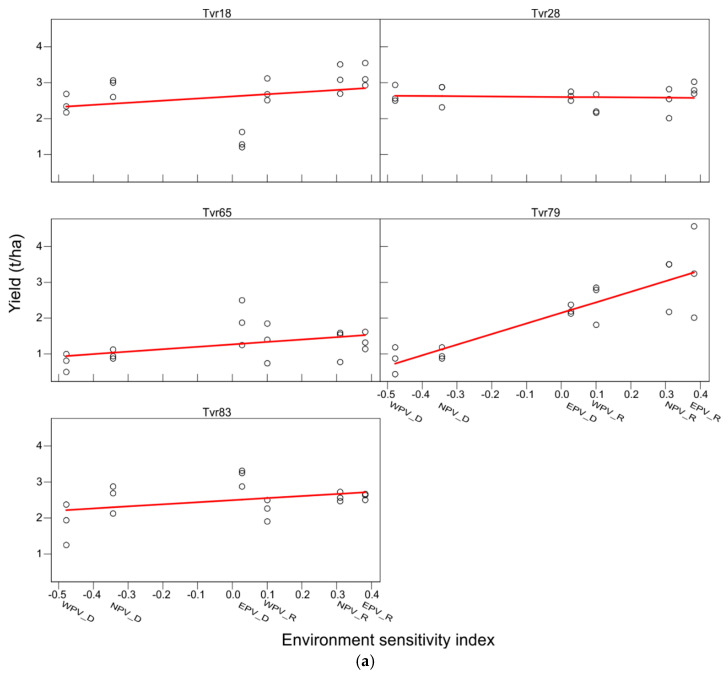

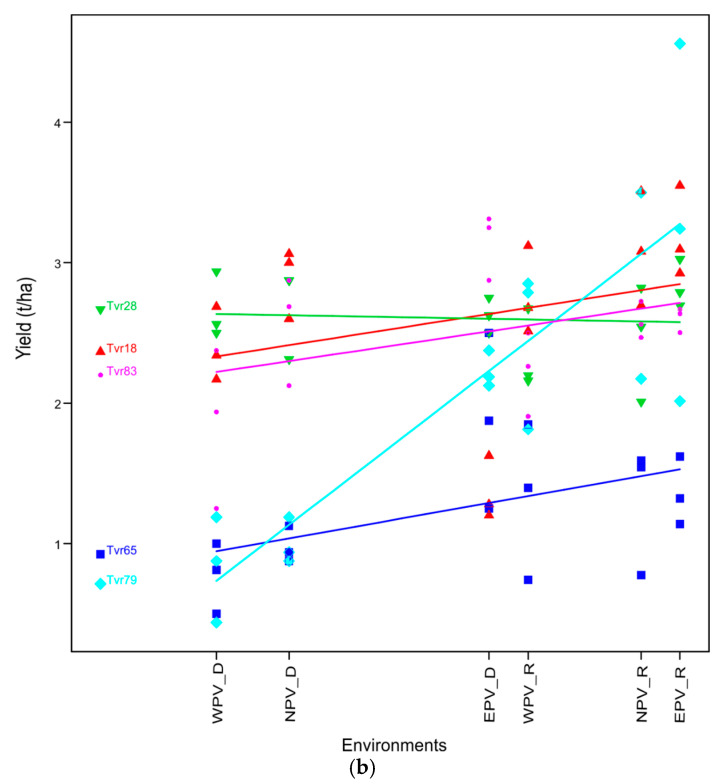
(**a**) Finlay and Wilkinson stability assessment of mungbean genotype across six APV environments. Negative values on the x-axis represent below-average environments; zero (0.0) represents average environment; and positive values represent above-average environments. WPV_D: East–West facing PV in the dry season; EPV_D: West–East facing PV in the dry season; NPV_D: no-PV shading in the dry season; WPV_R: East–West facing PV in the rainy season; EPV_R: West–East facing PV in the rainy season; NPV_R: no-PV shading in the rainy season. (**b**) Finlay and Wilkinson joint regression graph illustrating the nature and magnitude of the G × E interaction effect among five mungbean genotypes grown in six APV environments. WPV_D: East–West facing PV in the dry season; EPV_D: West–East facing PV in the dry season; NPV_D: no-PV shading in the dry season; WPV_R: East–West facing PV in the rainy season; EPV_R: West–East facing PV in the rainy season; NPV_R: no-PV shading in the rainy season.

The genotype and environment scores, sensitivity index, and ranks are shown in Table 4. The result showed that Tvr79 recorded the highest sensitivity index (2.95), followed by Tvr65 (0.68) and Tvr18 (0.60), and were ranked the least, whereas Tvr28 and Tvr83 had lower sensitivity scores (−0.07 and 0.57, respectively) and were ranked first and second, respectively. Likewise, the poorest (1.74) and most unstable environment was WPV-D (−0.48), while the EPV-R was the best (2.61) and most stable environment (0.38) and was ranked as 1 (Table 4). The response of each genotype across the APV environments is also shown in Figure 4a. The graph shows the regression line or line of best fit. A flat line implies the unresponsiveness of a genotype to environmental variations, and therefore high stability. A tilt to the vertical axis indicates high genotype sensitivity to environmental variations. Thus, the degree of tilt of a regression line towards the vertical axis reflects the degree of stability of the genotype. From the line of best fit in Figure 4a, Tvr28 and Tvr83 were the closest to a flat line and were the most stable genotypes. The mean response of each genotype across the six APV environments is illustrated in Figure 4b. The graph showed that the interaction effect was so strong as to cause a crossover effect or a change in ranking order of genotypes, which represents the strongest form of G × E interaction. As a component of total phenotypic variance, a large G × E interaction, especially of one leading to a crossing over effect, can negatively affect heritability and genetic advance. The greater the magnitude of the G × E interaction component, the smaller the heritability estimate and the lower the selection efficiency [34].

The objective of this study was to select genotypes with superior yield advantage and greater stability underneath the APV system. The study revealed that Tvr18 genotype recorded the highest mean but was unstable according to the IPCA2 (1.04), ASV (1.10), and the Finlay and Wilkinson sensitivity score (0.60) and rank (3rd) and is recommended for use in an APV system during the rainy season for which it showed superiority. Conversely, the genotypes Tvr28 and Tvr83, which demonstrated higher stability across the APV environments according to the IPCA1, IPCA2, ASV, and the Finlay and Wilkinson sensitivity scores (<0.60) and ranked second and third in seed yield after Tvr18, but first and second in terms of overall performance in yield and stability (Table 2 and Table 4), were recommended as the best mungbean genotypes for an Agri-PV system in a tropical climate setting. These genotypes are therefore strongly recommended for varietal development programs on the premise of their superiority in yield and broad adaptability across six contrasting APV environments in agreement with Lal et al. [35] and Ukwu et al. [36]. Correspondingly, the WPV-R and EPV-R environments were the ideal environments in this study.

### 2.3. Conclusions

Analyzing genotype by an environment interaction effect is crucial for the sustainability of APV systems globally because, selection gains can be negatively affected by a G × E interaction effect. Consequently, selecting genotypes that could consistently maintain high yield underneath the APV system is of utmost importance. The study objective was to select mungbean genotype(s) with high yield potential and broad adaptability across APV environments. Five mungbean genotypes, Tvr18, Tvr28, Tvr65, Tvr79, and Tvr 83, were assessed under three contrasting APV environments, EPV, NPV and WPV, in the rainy and dry seasons, cumulating to a total of six APV environments at the Agri-PV Food and Energy Training Center, University of Nigeria, Nsukka. Two G × E models, AMMI and the Finlay and Wilkinson joint regression analysis, confirmed significant genotype, environment, and G × E interaction effects for mungbean seed yield. Two genotypes, Tvr28 and Tvr83, displayed broad adaptability to the APV environments with higher yields (2.60 and 2.50 t ha^−1^), ranking first and second, respectively. In contrast, the Tvr79 genotype displayed the highest sensitivity (2.95) to environmental variation and was unstable across the environments with higher IPCA1 and ASV scores of −1.17 and 1.39, respectively. The EPV-R recorded the highest yield (2.61) with low interaction effect (0.38) whereas the WPV-D environment had the least yield (1.71) and was the most unstable (−0.48). This study recorded large G × E interaction effect which manifested in a change in ranking order of genotypes. Specifically, Tvr79 genotype ranked 1st in the NPV environment in the rainy season (3.08) and 6th in the NPV environment in the dry season. Overall, it ranked 2nd across the APV environments in the rainy season and 4th in the dry season. The implication of such a rank order change is enormous. A farmer who grew mungbean only in the NPV environment during the rainy season could conclude that Tvr79 genotype was the best. Such an erroneous conclusion could cost him his investment if he decides to grow only Tvr79 genotype in the dry season. Conclusively, the Tvr28 and Tvr83 genotypes and the EPV-R environment were the ideal genotypes and environment, respectively, and are therefore recommended for use in APV facilities in a tropical environment. Future APV research should explore G × E interaction analysis for other crops.

## 3. Materials and Methods

### 3.1. Experimental Site

The experiment was conducted at the Agri-PV Food and Energy Training Center, University of Nigeria, Nsukka, Enugu State, Nigeria (6°51′57″ N 7°24′57″ E) between December 2022 and March 2023 (dry season) and between April and July 2023 (rainy season). Nsukka has a mean annual rainfall of 1276 ± 706 mm, solar radiation of 1452 ± 269 w m^−2^, and temperature of 32 ± 5 °C [37]. Relative humidity varies and is influenced by seasons (rainy and dry seasons). The upper limit (about 89%) is usually experienced during the peak rainy season (July to August), while the lower limits (39–41%) occur during dry spells (December and January).

### 3.2. Experimental Materials and Design

Five mungbean genotypes, Tvr18, Tvr28, Tvr65, Tvr79, and Tvr83 (Table 5), sourced from the Department of Crop Science Genebank, University of Nigeria, Nsukka, after two generations of selection [30,31,32] for yield and adaptability, were grown under six environments [East–West facing PV in the rainy season (WPV-R), West–East facing PV in the rainy season (EPV-R), no-PV shading in the rainy season (NPV-R), East–West facing PV in the dry season (WPV-D), West–East facing PV in the dry season (EPV-D) and no-PV shading in Dry season (NPV-D)] at the Agri-PV Food and Energy Training Center, University of Nigeria, Nsukka. The experimental design was a split-plot design with the environment as the main plot treatment, and genotype as the sub-plot treatment replicated five times. A 20 kWp APV facility with dimensions 21 × 9.8 m in length and width was used for the study. The height of the APV facility ranged from its lowest point at 2.65 m to its apex at 3.15 m high, with a tilt angle of 20°. The APV facility was partitioned into three main plots (EPV, NPV, WPV), with the two main plots at the extreme fully covered (100% covered) with bifacial PV modules while the middle plot was covered with a transparent acrylic glass material (for unrestricted solar transmittance or full sunlight) to serve as the control.

### 3.3. Crop Establishment

Seeds were sown in 10 L pots prefilled with inert coconut fiber dust and placed at a spacing of 40 × 40 cm. Two seeds were sown per pot and later thinned down to one seed at one week after planting (WAP). Universol orange fertilizer (N-16%, P_2_O_5_-5%, K_2_O-25%, MgO-3.4%, Fe-0.10%, Mn-0.04%, B-0.01%, Cu-0.01%, Mo-0.001%, Zn-0.01%) sourced from ICL Holding Germany GmbH (67065 Ludwigshafenam Rhein) was applied at the rate of 2 g/L or 20 g per pot. Watering was carried out once daily according to the ET_Crop_ (3–5 mm).

### 3.4. Microclimate Variability of the Six APV Environments

**Figure 5:**
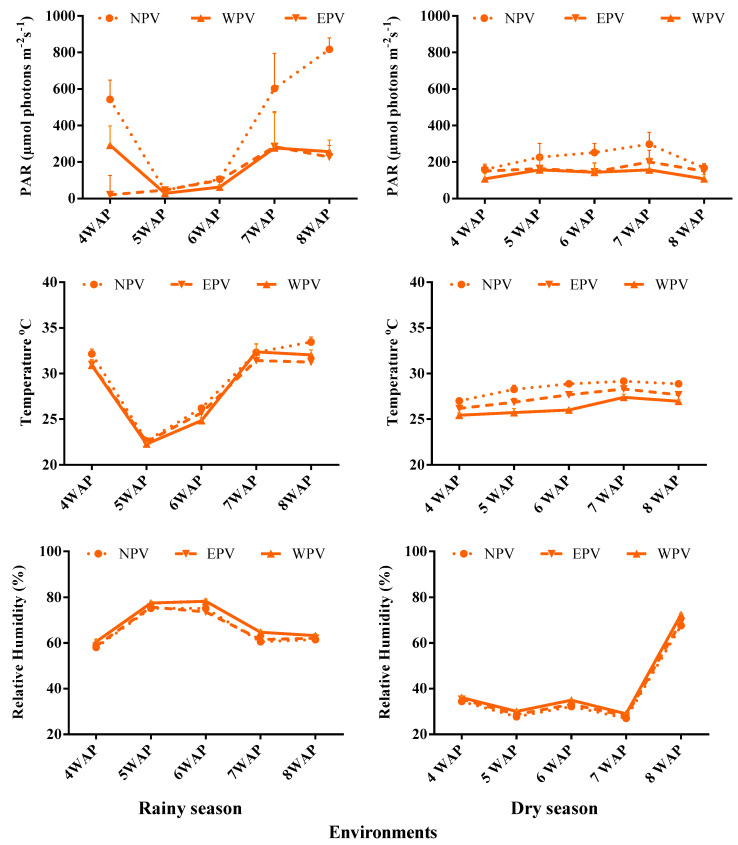
Variability in microclimate variables underneath three APV environments used for the study. WPV: East–West facing PV; EPV: West–East facing PV; NPV: no-PV shading.

The microclimate parameters of the six APV environments recorded weekly between 9 a.m. and 12 p.m. showed significant variation (*p* < 0.05). The NPV environments recorded higher photosynthetic active radiation (PAR) and temperature than the EPV and WPV environments, respectively, in both the dry and rainy seasons (Figure 5). Relative humidity on the other hand was higher underneath the WPV environments than the EPV and NPV environments for both seasons. Notably, PAR ranged from 107.8 to 157.0, 132.1–200.0, and 158.6–298.0 µmol photons m^−2^ s^−1^ underneath WPV, EPV, and NPV environments in dry season, and from 29.1 to 292.0, 20.6–284.7, and 45.1–817.0 µmol photons m^−2^ s^−1^, respectively, underneath the WPV, EPV, and NPV environments in rainy season. Variability in microclimate temperature ranged from 25.4 to 27.4, from 26.2 to 28.3, and from 27.0 to 29.2 °C underneath WPV, EPV, and NPV environments in the dry season, and from 22.3 to 32.4, from 22.5 to 31.4, and from 22.6 to 33.4 °C, respectively, underneath WPV, EPV, and NPV environments in the rainy season. Additionally, variation in relative humidity ranged from 29 to 72%, from 28 to 71%, and from 27 to 68% underneath WPV, EPV, and NPV, respectively, and from 61 to 78%, from 59 to 76%, and from 58 to 75%, respectively, underneath the WPV, EPV, and NPV environments. Data on microclimate variables were recorded for five weeks starting from the 4th week after planting using the Mini-PAM II device (WALZ, Effeltrich, Germany) according to the procedures used by Ukwu et al. [12].

### 3.5. Data Collection and Statistical Analysis

Data were collected from two middle row plants per treatment combination. Seed yield was calculated per plant and extrapolated to per hectare basis, following Ihejiofor et al. [38,39] and Ukwu et al. [12,30,31,32], as follows:

$$\mathrm{Seed}\;\mathrm{yield}\;(t\;{\mathrm{ha}}^{-1})=Sowing\;density\;\times \;seed\;weight\;per\;plant$$

The data were tested for significance using the additive main effect and multiplicative interaction (AMMI) analysis of variance procedure and the Finlay and Wilkinson joint regression approach which were able to partition the variation in the yield data set into components, accounting for both genotype and environment main treatment effects and the G × E interaction components [40]. The AMMI approach combines traditional ANOVA (Analysis of Variance) and PCA (Principal Component Analysis) into a single analysis framework that includes both additive and multiplicative effects [41].

The AMMI analysis followed a two-step process. First, standard ANOVA was used to estimate the main effects of genotype and environment. Then, PCA was applied to the interaction residuals. The AMMI model equation is as follows:

$$Y_{ij}=µ+G_{i}+E_{j}+\sum \lambda k\alpha ik\delta jk+R_{ij}+\varepsilon$$

where “Y_ij_ is the value of the ith genotype in the jth environment; μ is the grand mean; G_i_ is the deviation of the ith genotype from the grand mean; E_j_ is the deviation of the jth environment from the grand mean; λk is the singular value for PC axis k; αik and δjk are the PC scores for axis k of the ith genotype and jth environment, respectively; Rth is the residual and ε is the error term” [41]. AMMI’s stability value (ASV) was calculated following the formula proposed by Purchase [42], as follows:

$$ASV=\sqrt{[\left(\frac{SSIPCA1}{SSIPCA2}\right)\left(IPCA1SCORE\right){]}^{2}+(IPCASCORE2{)}^{2}}$$

where SSIPCA1/SSIPCA2 is the weight given to the IPCA1 value by dividing the IPCA1 SS by the IPCA2 SS, and the IPCA1 and IPCA2 scores are the genotypic scores in the AMMI model.

The GGE-biplot methodology, which is composed of two concepts, the biplot concept [43] and the GGE concept [44], were used to visually analyze the multi-environment yield trial (MEYTs) data. This methodology uses a biplot to show the factors (G and GE) that are important in genotype evaluation and that are also sources of variation in G × E analysis of MEYTs data [44,45]. Additionally, the GGE biplot analysis was employed to illustrate the “which-won-where” view and to identify the ideal genotype and environment by the average environment coordinates graphs. Finally, the Finlay and Wilkinson joint regression analysis [20] was also used to confirm the significance of the genotype and environment main effects, quantify the sensitivities of the genotypes to the different environments, illustrate the line of best fit for each genotype, and rank the genotypes based on yield performance and stability across different environments using the 18th edition of Genstat statistical software, version 18.1.0.17005. The Finlay and Wilkinson method uses regression to partition the G × E interaction term into linear and non-linear components based on the basic biometric genetic model as stated thus:

$$Y_{ij}=µ+{ɖ}_{i}+\left(1+\beta_{i}\right)e_{j}+\delta_{ij}+\varepsilon_{ij}$$

where µ is grand mean; ɖ_i_ is the effect of the ith genotype; e_j_ is the effect of the jth environment; β_i_ is the regression of (Y_ij_ − µ − ɖ_i_ − e_j_), i.e., g_ij_ on µ + e_j_; δ_ij_ is the deviation from regression for the ith genotype in the jth environment; and ε_ij_ is the random error.

## Figures and Tables

**Table 1:** AMMI analysis of variance.

Source	d.f.	s.s.	m.s.	%Total ss	%Treatment
Treatments	29	51.18	1.77 ***	82.5	
Genotypes	4	23.22	5.81 ***		45.4
Environments	5	8.55	1.71 ***		16.7
Block	12	1.39	0.12	2.2	
G × E Interaction	20	19.40	0.97 ***		37.9
IPCA 1	8	10.12	1.27 ***		19.8
IPCA 2	6	8.51	1.42 ***		16.6
Residuals	6	0.78	0.13		
Error	48	9.48	0.20	15.3	
Total	89	62.05	0.70		

d.f., degree of freedom; s.s., sum of squares; m.s., mean squares; *** Significant at 0.1% probability level.

**Table 2:** Yield scores of genotypes, environment, and G × E interaction; AMMI stability value (ASV) and IPCA scores of five mungbean genotypes grown in six different environments.

Genotype	EPV Dry	EPV Rainy	NPV Dry	NPV Rainy	WPV Dry	WPV Rainy	Mean	IPCA1	IPCA2	ASV
Tvr18	1.37	3.25	2.83	3.06	2.47	2.74	2.62	0.30	1.04	1.10
Tvr28	2.62	2.70	2.85	2.55	2.49	2.42	2.60	0.58	−0.07	0.69
Tvr65	1.89	1.47	1.05	1.33	0.73	1.15	1.27	0.08	−0.52	0.53
Tvr79	2.23	3.23	1.03	3.08	0.79	2.52	2.15	−1.17	0.10	1.39
Tvr83	3.14	2.62	2.37	2.48	2.04	2.33	2.50	0.21	−0.55	0.61
Mean	2.25	2.65	2.02	2.50	1.71	2.23	2.23			
IPCA1	−0.15	−0.53	0.79	−0.54	0.72	−0.30				
IPCA2	−1.17	0.35	0.16	0.32	0.15	0.20				

EPV: West–East facing PV; NPV: no PV shading; WPV: East–West facing PV; IPCA: interaction principal component axis; scores are in t ha^−1^.

**Table 3:** Finlay and Wilkinson joint regression analysis of variance.

Source	d.f.	s.s.	m.s.	% of Total ss
Genotypes	4	23.22	5.81 ***	37.42
Environments	5	8.55	1.71 ***	13.78
Sensitivities	4	9.20	2.30 ***	14.83
Residual	76	21.07	0.28	
Total	89	62.05	0.70	

d.f., degree of freedom; s.s., sum of squares; m.s., mean squares; *** significant at *p* < 0.0001.

**Table 4:** Finlay and Wilkinson joint regression analysis showing genotype and environment scores, sensitivity index, and rank.

Treatment	Mean (t ha^−1^)	Sensitivity	Rank
Genotype			
Tvr18	2.62	0.60	3
Tvr28	2.60	−0.07	1
Tvr65	1.27	0.68	4
Tvr79	2.15	2.95	5
Tvr83	2.50	0.57	2
Environment			
EPV-D	2.25	0.03	4
EPV-R	2.61	0.38	1
NPV-D	1.88	−0.34	5
NPV-R	2.54	0.31	2
WPV-D	1.75	−0.48	6
WPV-R	2.33	0.10	3

EPV: West–East facing PV; NPV: no PV shading; WPV: East–West facing PV; -D: dry season; -R: rainy season.

**Table 5:** Morphological characteristics of five mungbean genotypes used for the study.

Genotype	Growth Pattern	Hypo-Cotyl Color	Terminal Leaf Shape	Leaf Color	Petiole Color	Petiole Length	Seed Shape	Seed Luster
Tvr18	Erect	Greenish purple	Cuneate	Green	GP	Long	Round	Dull
Tvr28	Erect	Greenish Purple	Cuneate	Green	GP	Medium	Round	Dull
Tvr65	Erect	Greenish purple	Cuneate	Green	GP	Medium	Round	Dull
Tvr79	Semi- erect	Purple	Cuneate	Green	GP	Medium	Round	Dull
Tvr83	Spreading	Green	Ovate	Dark-Green	Purple	Short	Oval	Shiny

GP: greenish-purple [30].

## Data Availability

Data are contained within the article.
